# Weight change in control group participants in behavioural weight loss interventions: a systematic review and meta-regression study

**DOI:** 10.1186/1471-2288-12-120

**Published:** 2012-08-08

**Authors:** Lauren Waters, Alexis St George, Tien Chey, Adrian Bauman

**Affiliations:** 1 Cancer Prevention Research Centre, School of Population Health, The University of Queensland, Herston, Brisbane, QLD, 4006, Australia; 2 Prevention Research Collaboration, School of Public Health, The University of Sydney, Sydney, NSW, 2006, Australia

## Abstract

**Background:**

Unanticipated control group improvements have been observed in intervention trials targeting various health behaviours. This phenomenon has not been studied in the context of behavioural weight loss intervention trials. The purpose of this study is to conduct a systematic review and meta-regression of behavioural weight loss interventions to quantify control group weight change, and relate the size of this effect to specific trial and sample characteristics.

**Methods:**

Database searches identified reports of intervention trials meeting the inclusion criteria. Data on control group weight change and possible explanatory factors were abstracted and analysed descriptively and quantitatively.

**Results:**

85 trials were reviewed and 72 were included in the meta-regression. While there was no change in control group weight, control groups receiving usual care lost 1 kg more than control groups that received no intervention, beyond measurement.

**Conclusions:**

There are several possible explanations why control group changes occur in intervention trials targeting other behaviours, but not for weight loss. Control group participation may prevent weight gain, although more research is needed to confirm this hypothesis.

## Background

The increasing prevalence of overweight and obesity has become a cause for concern over the past decade [1] with overweight and obesity being major determinants of a number of chronic health conditions including hypertension, cardiovascular disease, diabetes and cancer [2]. The importance of implementing interventions to address this public health problem has been recognised by the World Health Organization [1,3] and the US Surgeon General [4]. Consequently, there is now an extensive body of literature addressing the efficacy of behavioural approaches to obesity prevention and treatment through encouraging increased physical activity and reduced energy intake. Such research is usually conducted as randomized trials, where participants receiving an intervention are compared to a control group who receive no treatment or current best practice (usual care) [5,6].

Unanticipated improvements in the behaviour of control group participants have been observed in intervention trials targeting an array of behaviours, including hazardous drinking [7], antiretroviral therapy adherence [8], physical activity [9], and chronic disease self-management [10]. Such improvements may occur in response to undergoing baseline assessment; due to participants’ awareness of being involved in an experimental trial; or due to the delivery of more intensive ‘usual care’ than would be encountered beyond the research context [11]. It is possible that control group improvements may also occur in behavioural weight loss intervention trials. Control group improvements have the potential to reduce or even nullify intervention effects through reducing statistical power to detect significant effects. Therefore, understanding when and why control group improvements occur has important implications for researchers, not only with respect to the interpretation of intervention effects, but also in relation to the design of future behavioural weight loss intervention trials.

Reviews of the behavioural weight loss intervention literature have alluded to the fact control group participants may lose weight through participating in an intervention trial [12]. However, there have been no systematic investigations specifically addressing weight change in control group participants in behavioural weight loss intervention trials. Nor has an attempt been made to quantify the amount of weight change that can be expected for participants *not* receiving the intervention; or to identify factors that are likely to predict a greater amount of weight change (particularly weight loss) among control group participants. The first objective of this study is to systematically review the behavioural weight loss intervention literature in order to describe the associations between control group weight change and specific trial and sample characteristics. The second objective is to conduct a meta-analysis to quantify the amount of weight change that occurs only in control groups, and a meta-regression analysis to relate the size of this effect to specific trial and sample characteristics. This meta-analysis will not compare intervention group effect sizes with control group effect sizes as the aim of this study is not to draw conclusions on the effectiveness of behavioural interventions in achieving weight loss, but to examine the amount of weight change that occurs solely due to (control group) participation in such trials.

## Methods

### Criteria for inclusion

Reports of randomised controlled trials or quasi-experimental trials evaluating behavioural weight loss interventions targeting adult participants, and that were published in English between 2005 and 2010 were potentially eligible for inclusion in this review. An extensive number of behavioural weight loss interventions have been published and the decision to limit the review to papers that were published within this time frame was guided by practical considerations.

Intervention trials were excluded from the review if the primary objective of the trial was not weight reduction; or if they implemented strategies other than behaviour change in order to achieve weight reduction (e.g., intervention components included pharmacological therapy, nutritional supplementation, herbal remedies or surgery). Trials were also excluded if they did not recruit a control or usual care group, or if participants allocated to the comparison condition received an alternative behavioural weight loss intervention or an attention control condition. Trials that did not report, or provide sufficient information to enable calculation of weight change (in kilograms) from baseline to follow up for each group were also excluded. Finally, trials recruiting participants who were pregnant or lactating, or had a medical condition which could confound the effect of a weight loss intervention (e.g., Prader Willi syndrome) were also excluded. No restriction was placed on the duration of trials; however, for trials that reported outcomes post-intervention and following a period of maintenance where no intervention was delivered, weight changes reported immediately following the intervention (not following the maintenance period) were used. This was done in order to standardise the definition of intervention duration for trials with and without a maintenance period.

### Electronic searches

Electronic databases (Pub Med [1951], Web of Science [1981], EMBASE [1980], PsychINFO [1967]) were searched in December 2010 for reports of weight loss intervention trials using a combination of the following Medical Subject Headings (MeSH) and text words: (((obes* OR overweight OR weight gain* OR weight los*OR weight cycling OR weight reduc* OR weight maint*OR weight decreas* OR body mass index OR adipos* OR overload syndrom* OR weight watch* OR weight control*) AND (exercise* OR exercise-therapy OR physical education* OR physical fitness OR exertion* OR sport* OR walking OR jogging OR swimming OR bicyc* OR cycling OR weight lift* OR gymnastic* OR danc* OR strength train* OR resistance train*OR aerobic train* OR Lifestyle OR Health* educ* OR health* behav* OR health* promot*OR nutrition* OR diet* OR diet therapy OR diabetic diet) AND (random* OR clinical trial OR control group OR meta analysis OR intervention OR random allocation OR intervention studies))).

### Study selection

One person, LW, screened the titles and abstracts of articles identified through electronic database searches. Full text versions of reports of trials that appeared to be relevant to the review were retrieved and read in full. A checklist developed prior to the search, and based on the list of inclusion and exclusion criteria, was used to systematically identify papers for inclusion. A second person (AS) independently screened 15% of the articles (selected at random) and agreement between reviewers was 100%.

### Data abstraction

Data was abstracted and coded by two reviewers (LW and AS). The primary outcome variable was mean weight change in kilograms from baseline to post-intervention follow up. Where this variable was not reported, but the mean weight for each group at baseline and follow up was given, change was calculated. For trials that reported weight in pounds, a conversion to kilograms was undertaken. Information on characteristics that were hypothesised to be potentially associated with control group weight change was recorded, and categorised as being related to trial design, treatment of the control group, or characteristics of the enrolled participants.

#### Trial design and methodology

Weight loss interventions were evaluated using randomised controlled trials or quasi-experimental (non-randomised controlled) trials. Participant recruitment strategies were categorised as being either an approach that involved identifying potential participants through searches of existing registers (e.g., general practice lists or existing trial cohorts) or appealing to volunteers through the media or by contacting existing community organisations (such as schools, workplaces or churches). Trial duration was defined as the length of time from baseline to immediate post-intervention follow up (excluding any period of maintenance). Sample size and the number of times a participant underwent assessment of body weight were also recorded.

#### Control group treatment

The description of the treatment delivered to the control group was categorised as being a “no intervention control” condition, “waiting list control” condition or “usual care”. To be categorised as a no intervention control group, participants allocated to this group must have received no treatment other than undergoing assessments. Similarly, participants in a waiting list control group received no treatment during the trial, but were informed that they would have the opportunity to receive the intervention components following the completion of the study. Participants allocated to a usual care control group received the same level of treatment that a person would normally have received, or could have had access to, outside of the intervention context. Usual care treatment may have included the issue of standard print leaflets addressing topics related to diet or physical activity, brief education sessions addressing topics such as risk factors for chronic disease, or advice to maintain usual behaviour patterns.

#### Characteristics of participants

Information on participants’ baseline characteristics, including age, gender, health status and body mass index, were abstracted and coded. The mean age of the sample in each trial was recorded, as was the proportions of female participants. Baseline health status was determined in the following way: participants in trials that specifically aimed to recruit people with an existing medical or psychiatric condition (e.g., cancer, diabetes, chronic heart disease, schizophrenia, obstructive sleep apnoea) were defined as having a chronic disease. In trials where recruitment was not targeted towards people with an existing medical condition, but where the mean BMI of the sample was in the overweight or obese range (BMI > 25 kg/m^2^) [13], participants were categorised as being at risk of a chronic disease. For trials where the mean BMI of the sample was in the healthy weight range (BMI ≥ 18 kg/m^2^ and ≤ 25 kg/m^2^) and participants were free from existing medical and psychiatric conditions, participants were classified as being recruited from a healthy population.

### Data analysis

#### Bivariate analysis

A simple, descriptive bivariate analysis was conducted to assess the associations between control group weight change in kilograms, and variables hypothesized to be potentially associated with control group change (PASW v.18; SPSS, Chicago, IL). The dependent variable (control group weight change) was not normally distributed; therefore non-parametric tests of association were employed (Mann–Whitney U test or Kruskal-Wallis test for categorical variables and Spearman rank correlation coefficients for continuous variables). Continuous variables (trial duration, number of assessments undertaken, mean age and BMI in each trial) were also transformed into categorical values according to the median value, or another logical value to avoid loss of statistical power in subsequent analyses as these variables were not normally distributed and the range of values were small and inconsistent across all studies.

#### Meta-analysis and meta-regression

The study protocol adhered to PRISMA guidelines for reporting systematic reviews and meta-analyses. The meta-analysis and meta-regression analysis were performed using STATA v.11 (StataCorp, 2009). Publication bias was investigated visually using a funnel plot and formally by Egger’s and Begg’s tests, using the user-written “meta” and “metabias” commands. The percentage of variation attributable to heterogeneity (I^2^) was computed from Cochran Q statistic. Given the potential for heterogeneity in the studies reviewed, a random effects model was deemed to be most appropriate. The standard error (SE) of the primary outcome variable (weight change in kilograms) was extracted directly from published reports, or computed from the reported standard deviation (SD), 95% confidence limits or exact p-value. For trials that did not report mean weight change, but reported mean group weight (and either SE or SD) at baseline and follow up, standard errors of weight change were calculated assuming an intra-correlation coefficient of ρ = 0.5. P values that were reported as <0.01 or <0.0001 were replaced by 0.005 and 0.00005 respectively. When the upper bound <0.01 and <0.0001 were not provided; we took the mid-point of 0.01 to 0.001 and 0.0001 to 0.00001.

Meta regression (“metareg” command) was performed to investigate the association between weight change and trial and sample characteristics that were designated *a priori*. The characteristics under investigation were those included in the bivariate analysis, as well as trial sample size (n ≥100 or <100) and the method by which the SE of weight change was determined (reported or calculated based on SE/SD/exact p-values). Dichotomised categorical versions of continuous variables were used in the meta-regression analysis to prevent loss of statistical power. Uni-variable and multi-variable meta-regression models were carried out. Results are presented as random effect coefficient estimates with 95% confidence intervals (CI) and p-values.

## Results

**Figure 1:**
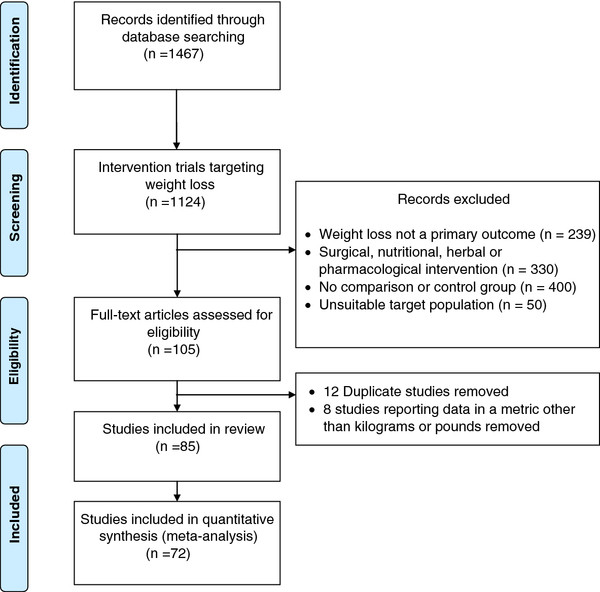
Flow diagram of studies included in the review.

Following the removal of duplicates, the total number of references identified through electronic database searching was 1,467; of which 1,124 were intervention trials targeting weight loss. Reports of trials in which weight change was a secondary, and not primary, outcome were excluded (n = 239); as were studies that reported on the evaluation of the effectiveness of a surgical procedure, nutritional or herbal supplement, or pharmacological treatment (n = 330). Reports of trials without a control or usual care comparison group (n = 400), and where recruited participants were pregnant, lactating or had a medical condition that could influence their ability to lose weight (n = 50) were also excluded. The remaining 105 publications reported the outcomes from 93 individual behavioural weight loss intervention trials. A further 8 of these were excluded because weight change was reported in a metric other than kilograms or pounds (e.g., BMI). Therefore, eighty-five reports of intervention trials met the inclusion criteria for this review. Seventy-two of these reports provided either the SE of weight change for the control group, or sufficient information to allow for this to be calculated (SE/SD/exact p-value of control group weight at baseline and follow-up) and were therefore included in the meta-regression analysis (Figure 1). A full list of the intervention trials included in the review is provided in Additional file 1: Appendix 1.

### Description of studies included in the review

#### Trial design and methodology

Sample sizes for the 85 trials included in the review ranged from 15 [14] to 68,591 [15] with a median sample size of 81 participants. The majority of trials (n = 78; 92%) employed a randomized controlled trial design with the remaining seven (8%) being quasi-experimental trials. Detailed accounts of the strategies used to recruit participants were provided in 76 reports (89%), with over half of those trials (n = 49; 58%) recruiting participants through appeals to volunteers via the media or by contacting existing community organisations (such as schools, workplaces or churches). The remainder (n = 36; 42%) identified participants from existing registers (e.g., general practice lists or existing trial cohorts). The duration between baseline and post-intervention follow up spanned from six weeks [16] to four and a half years, [17] with the median duration being 6 months. Control group participants in all trials were assessed at baseline and follow up; and at least one additional assessment was conducted in almost one third of studies (n = 26, 31%). In all but one trial [18], weight was measured objectively in a clinical setting.

#### Control group treatment

In 33 trials (39%), participants were allocated to a no intervention control condition and received no treatment other than undergoing assessment. In a further 14 studies (16%), participants in the control group were informed that they would receive the intervention at the completion of the trial (wait list control). Participants in the comparison group in the remaining 38 studies (44%) received usual care. The content of the usual care treatment was not consistently described, but most often involved the receipt of standard off-the-shelf print material addressing health behaviours, but may have also included a single information session delivered in a group setting or individual consultation.

#### Characteristics of participants

The mean age of the study sample was reported for 81 trials and varied from 19.2 years [19] to 63.7 years [20]. For those 81 trials, the median of the distribution of sample ages was 48.7 years. In terms of the gender distribution within study samples, 24 trials (28%) recruited only females, and a further 24 trials (28%) recruited mostly females (≥60% female). In contrast, only seven trials (8%) exclusively targeted male participants, while in a further eight trials (9%) males formed more than 60% of the sample. Twenty-two trials (26%) had a relatively even gender distribution (40-60% female). Eighty-one studies reported the mean BMI for the control group at baseline, and this value varied from 23.8 kg/m^2^[21] to 43.6 kg/m^2^[22]. The median of the distribution of BMIs of the control group samples was 31.1 kg/m^2^. In three trials (4%), the mean BMI for the control group was in the healthy weight range, while in the remainder of studies, the control group sample was either overweight (n = 27; 33%) or obese (n = 48, 59%). Of the three studies where the mean BMI of the control group was in the healthy weight range, one targeted participants who were at high risk of gaining weight, one recruited East Asian males who met the criteria for overweight when ethnicity-specific cut-points were used and the remaining study was a family-based study. The majority of studies targeted participants who were at risk of a chronic disease through being overweight or obese, but who were otherwise healthy (n = 55, 65%). Almost one third of studies (n = 26, 31%) recruited participants who had been diagnosed with a chronic disease, and a small minority of studies (n = 4; 5%) targeted healthy populations.

### Bivariate analyses

Across all 85 trials, the median weight change for control groups was negligible (median = +0.1 kg; IQR = −0.85 kg to +0.8 kg), and ranged from a mean group weight loss of 5.8 kg [23] to a mean group weight gain of 4 kg [24]. Statistical analyses revealed no significant associations between control group weight change and the explanatory variables (Table 1). While the purpose of this review was not to quantify the amount of weight change in intervention groups, or to examine factors associated with greater weight loss among participants who received an intervention, analyses were repeated for the intervention groups for comparative purposes to demonstrate differences in the average weight change between intervention recipients and controls. The median weight loss for the intervention groups across all trials was −2.7 kg (IQR = −5 kg to −1.35 kg; range: -18.7 kg [25] to + 2.5 kg [26]). 

**Table 1:** Bivariate associations between explanatory variables and control group weight change (kg)

**Trial and participant characteristics:**	**Study n**	**Median group weight change (Inter-quartile range)**		**p-value**
Trial Characteristics				
Trial design				
* Randomised controlled trial*	78	0.0	(−1.1, 0.8)	
* Quasi-experimental study design*	7	0.5	(−0.2, 0.9)	0.200
Recruitment strategy				
* Searches of existing registers*	36	0.4	(−0.8, 1.25)	
* Media appeal for volunteers*	40	0.0	(−1.1, 0.6)	0.498
Intervention duration*^δ^				
* ≤6 months*	54	0.1	(−0.8, 0.6)	
* >6 months*	31	0.2	(−1.1, 1.9)	0.637
Total number of assessments*				
* 2 (baseline and follow up)*	59	0.2	(−0.5, 0.7)	
* >2 (at least one interim assessment)*	26	−0.6	(−1.4, 1.3)	0.468
Control group treatment				
* No intervention control group*	33	0.3	(−0.4, 0.6)	
* Wait list control group*	14	0.4	(−0.4, 2.1)	
* Usual care group*	38	−0.5	(−1.3, 0.8 )	0.139
Sample Characteristics				
Age*				
* ≤50 years*	45	0.4	(−1.1, 0.9)	
* >50 years*	36	0.0	(−0.7, 0.8)	0.427
Gender				
* Mostly female (>60 % female)*	48	−0.1	(−1.1, 0.8)	
* Mixed gender or mostly male*	37	0.3	(−0.8, 1.0)	0.346
Target population				
* Healthy or at risk of chronic disease*	59	0.0	(−1.1, 0.6)	
* Chronic disease*	26	0.3	(−0.8, 1.2)	0.247
Baseline mean BMI category				
* Healthy or overweight (≥ 18 - <25 kg/m*^*2*^*)*	33	0.3	(−0.5, 0.8)	
* Obese (> 30 kg/m2)*	48	0.0	(−1.1, 0.9)	0.441

*Dichotomised based on the median value.

^δ^ When analysed as a continuous variable, there was no difference in trial duration for studies that had a mean control group weight loss and those that had a mean control group weight gain.

### Meta-analysis and meta-regression

**Figure 2:**
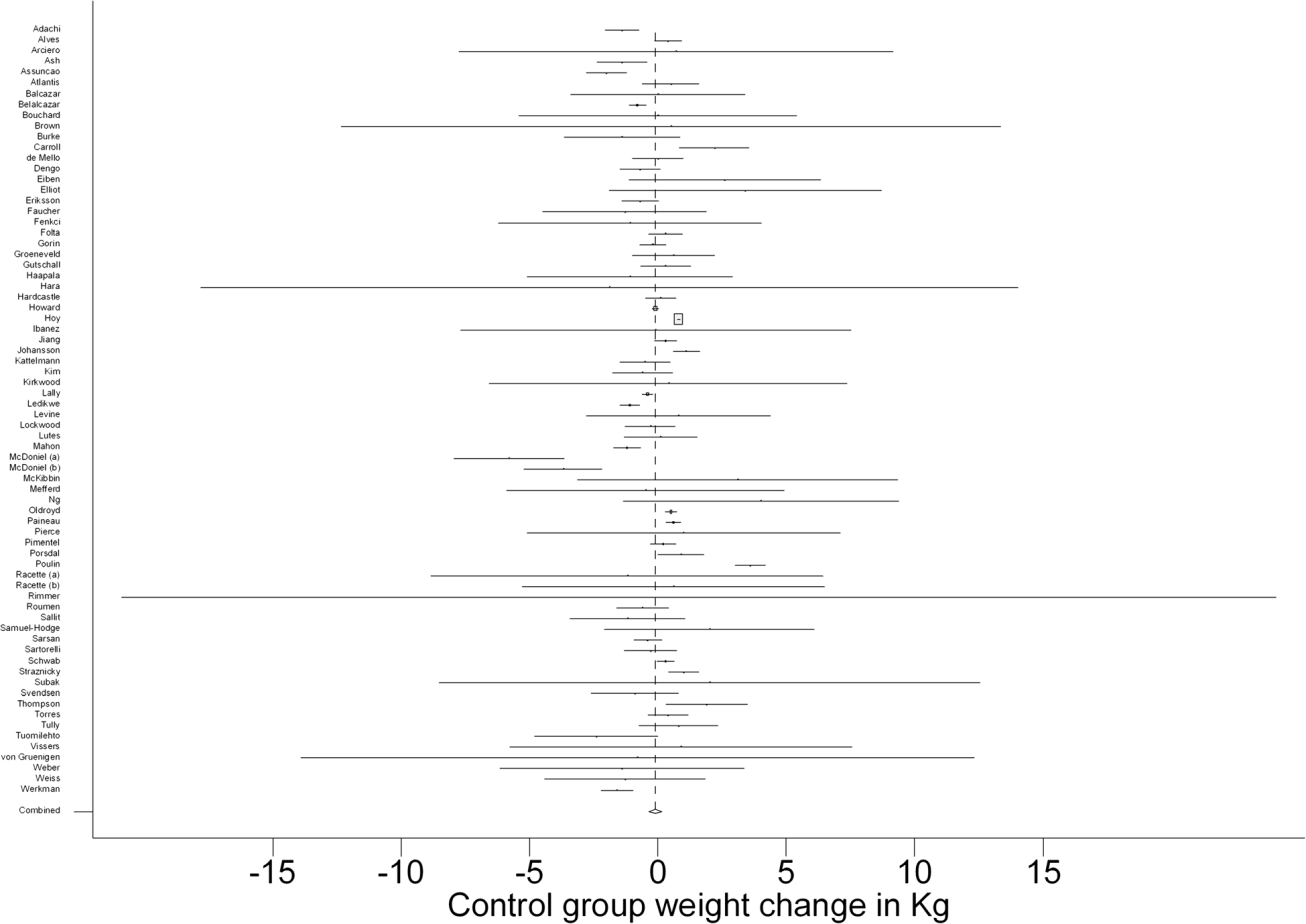
Forest plot showing control group weight change (n = 72).

A high level of heterogeneity was present (Q = 847.1; df = 71; p = 0.000) with the I^2^ statistic indicating that 92% of the variation in control group weight change was attributable to heterogeneity in the studies reviewed. The results from the meta-analysis confirmed the findings of the bivariate analysis. The random effect combined weight change for the control group was −0.1 kg (95%CI: -0.4, 0.1) and not statistically significantly different from zero (Figure 2).

In the uni-variable meta-regression model, three variables were found to be significant predictors of control group weight change (Table 2). The meta-regression coefficient for weight change was significantly lower in trials with a usual care group compared to those with a no intervention control group (−0.84 kg; CI:-1.55, -0.13; p = 0.021); in randomised controlled trials compared to quasi-experimental trials (−1.11 kg; CI: -2.17, -0.06; p = 0.038) and in trials where the SE of weight change was derived from the SE/SD/exact p-value (−1.02 kg; CI: -2.00, -0.03; p = 0.043) as opposed to other methods. In the multi-variable regression model, the only significant predictor of control group weight change was control group treatment with trials with a usual care group having a significantly lower meta-regression coefficient for weight change than trials with a no intervention control group (−1.23 kg; CI: -2.22, -0.23; p = 0.016) (Table 2).

**Table 2:** Random effects meta-regression analysis, association of explanatory variables with control group weight change (kg)

		**Uni-variable**			**Multi-variable**		
**Trial and participant characteristics:**	**Study n**	**Weight change coefficient (95%CI)**		**p-value**	**Weight change coefficient (95%CI)**		**p-value**
Method of ascertaining SE of weight change							
* Reported (ref = 0)*	23						
* From SE/SD/exact p*	49	−1.02	(−2.00, -0.03)	0.043	−0.82	(−1.85, 0.21)	0.119
Sample size							
* <100 (ref = 0)*	61						
* ≥100*	11	−0.01	(−0.85, 0.83)	0.976	0.68	(−0.32, 1.68)	0.181
Trial design							
* Quasi-experimental (ref = 0)*	6						
* RCT*	66	−1.11	(−2.17, -0.06)	0.038	−0.37	(−1.56, 0.81)	0.538
Recruitment strategy							
* Media appeal for volunteers (ref = 0)*	43						
* Searches of existing registers*	29	−0.03	(−0.71, 0.66)	0.937	−0.13	(−0.62, 0.88)	0.732
Intervention duration							
* ≤6 months (ref = 0)*	47						
* >6 months*	25	0.29	(−0.41, 1.00)	0.412	0.72	(−0.17, 1.61)	0.113
Total number of assessments							
* 2*	52						
* >2 (ref = 0)*	20	−0.47	(−1.24, 0.30)	0.234	−0.27	(−1.06, 0.51)	0.492
Control group treatment							
* No intervention control group (ref = 0)*	26						
* Wait list control group*	13	0.26	(−0.65, 1.16)	0.579	0.85	(−0.10, 1.80)	0.079
* Usual care group*	33	−0.84	(−1.55, -0.13)	0.021	−1.23	(−2.22, -0.23)	0.016
Age							
* ≤50 years (ref = 0)*	34						
* >50 years*	35	−0.01	(−0.71, 0.69)	0.971	−0.32	(−1.07, 0.43)	0.406
Gender							
* Mixed gender or mostly male (ref = 0)*	33						
* Mostly female (>60 % female)*	39	−0.22	(−0.90, 0.45)	0.517	0.14	(−0.56, 0.84)	0.695
Target population							
* Chronic disease (ref = 0)*	23						
* Healthy or at risk of chronic disease*	49	−0.38	(−1.09, 0.32)	0.286	−0.49	(−1.26, 0.29)	0.216
Baseline BMI category							
* Healthy or overweight (≤ 30 kg/m2) (ref = 0)*	26						
* Obese (> 30 kg/m2)*	43	−0.24	(−0.96, 0.48)	0.513	−0.24	(−0.95, 0.47)	0.501

In comparison, the random effect combined weight change for the intervention group was −3.36 kg (95% CI:-3.89, -2.82), and significantly different from zero. In the uni-variable model, the meta-regression coefficient for weight change was significantly higher in trials with a sample size ≥100 (1.95 kg; CI: 0.07, 3.82; p = 0.042), but none of the explanatory variables were significant predictors of intervention group weight change in the multi-variable model (Table 2).

## Discussion

This research is novel in providing an understanding of changes in the behaviour of control groups, rather than intervention groups. This is the first review to investigate weight change occurring in control groups in behavioural weight loss intervention trials, and whether certain trial or sample characteristics might predict greater control group weight change. The overall weight change in control groups was not significantly different from zero; however, there is some evidence that providing usual care to control groups may have a greater effect on weight loss than if no treatment were provided. Control groups receiving usual care lost significantly more weight than no intervention control groups (1.23 kg weight loss compared to no weight change respectively).

Favourable behaviour changes among control group participants have been observed for a diverse range of other health behaviours [7-9]. The fact that these findings were not replicated in the sample of weight loss intervention trials reviewed is an interesting finding. There are several plausible explanations for why control group improvements may be observed in intervention trials targeting other behaviours, but not those that aim to change participants’ body weight through employing behaviour change strategies.

Behavioural weight loss intervention trials are distinct from those targeting other behaviours in that the primary outcome is most often physical (i.e., change in body weight) rather than behavioural (i.e., change in diet or physical activity). Furthermore, body weight is objectively assessed using calibrated scales, as it was in all but one of the intervention trials reviewed. In contrast, while objective measures are available for measuring physical activity and alcohol consumption (two behaviours for which control group improvements in intervention trials have been reported), there is a greater reliance on self-report measures of behaviour change [7,9]. Self-report measures are susceptible to social desirability bias [27] and it may be that self-reported behaviour changes in control groups reflect unreliable reporting rather than actual behaviour changes. Alternatively, it may be that control group participants in behavioural weight loss intervention trials do change their behaviour, but not sufficient to induce actual weight loss.

Given multiple, inter-related biologic, genetic, social, and cultural influences, it is difficult to induce behaviour changes that lead to sustained weight loss [28,29]. Thus, it is possible that the lack of control group weight loss observed in this review reflects both the complexity and difficulty in achieving weight loss through behavioural strategies alone [12]. Control group changes may be more likely in intervention trials targeting less complex behaviours. For example, control group changes in behavior and objectively measured clinical outcomes have been seen in intervention trials targeting antiretroviral medication adherence [8,30]. The evidence in this field of research indicates an association between the quality of usual care treatment and control group outcomes [30].

While the findings of this review appear to suggest that neither the treatment of the control group, nor the application of procedures associated with administering an intervention trial (e.g., measurement, recruitment strategies) appear sufficient to significantly change participants’ body weight, it is possible that control group participation confers benefits through the prevention of weight gain. Interventions aimed at preventing weight gain in healthy populations have become the focus of research attention in recent years [31,32]. Prospective studies show that there is a tendency towards weight gain over time [33,34]. Although the time period considered in the studies reviewed was likely to be too short to detect control group weight gain, if weight gain prevention occurred, there would be implications for the development of minimal intensity weight gain prevention interventions. Further research dedicated to understanding which elements of the control group assessment might be useful for weight gain prevention (research procedures, the content of usual care, or the interaction between the two) is warranted.

This review has a number of limitations. First, the results of the meta-analysis must be interpreted with caution, given the extent of heterogeneity observed. This review was limited to intervention trials published between 2005 and 2010. The decision to limit the review to papers published within this timeframe was made for practical purposes as the literature addressing behavioural weight loss interventions is vast. It is possible that the results of this study may have been impacted by the decision to limit the review to this timeframe. It is also not possible to exclude publication bias given the reliance on published manuscripts. A large number of explanatory variables were included in the meta-regression model, potentially increasing the probability of false positive conclusions. However, this is unlikely to have been a problem, given that very few of the explanatory variables were significantly associated with the outcome variable. A more parsimonious model containing a fewer explanatory variables yielded similar results. The usual care treatment delivered to participants in the control group was often not described comprehensively, precluding analysis of the effects of the content of usual care treatment on control group outcomes. The absence of sufficient information to enable replication of the usual care condition has been noted in previous studies and is a barrier to understanding control group improvements [8,30,35]. However, given the overall null findings related to weight change, the utility of such an analysis would be questionable in the context of this review.

## Conclusions

Contrary to other areas of behaviour change research where control group improvements have been observed [7-9], in behavioural weight loss interventions control groups mostly showed negligible changes in weight status. The findings of this review suggest that it is difficult to achieve weight loss, and exposure to measurement alone is insufficient to initiate measureable behaviour change. Minimal intensity interventions may have a role in a range of health behaviours but appear not to do so with respect to weight loss.

## Competing interests

The authors declare that they have no competing interests.

## Authors’ contributions

All four authors contributed to the conception and design of the study. LW carried out the literature search. LW and ASG reviewed articles for inclusion in the review and abstracted data. LW conducted the descriptive data analysis and TC completed the meta-regression analysis. All authors helped to draft the manuscript and all authors read and approved the final manuscript.

## Pre-publication history

The pre-publication history for this paper can be accessed here:

http://www.biomedcentral.com/1471-2288/12/120/prepub

## Supplementary Material

Additional file 1 ** Appendix. **Description of intervention trials included in “Weight change in the control group participants in behavioural weight loss interventions: A systematic review and meta-regression study [14-26,36-107]. Click here for file
