# Supplementary material for: Weight change in control group participants in behavioural weight loss interventions: a systematic review and meta-regression study
Source: BMC Med Res Methodol. 2012 Aug 8;12:120. doi: 10.1186/1471-2288-12-120 (PMC3499351; doi:10.1186/1471-2288-12-120)
Supplement: Additional file 1 — Appendix. Description of intervention trials included in “Weight change in the control group participants in behavioural weight loss interventions: A systematic review and meta-regression study [14-26,36-107]. [file 1471-2288-12-120-S1.docx]

**Additional File 1 – Appendix: Description of intervention trials included in “Weight change in the control group participants in behavioural weight loss interventions: A systematic review and meta-regression study.**

| **First Author** | **Year** | **Control group (n)** | **Control treatment** | **Trial duration (months)** | **Control group baseline BMI (kg/m^2^)** |
| --- | --- | --- | --- | --- | --- |
| Adachi, Y ^36^ | 2007 | 50 | Usual care | 7 | 26.1 |
| Alves, JG ^37^ | 2009 | 78 | No intervention control | 6 | 30.3 |
| Arciero, PJ ^38^ | 2006 | 17 | No intervention control | 3 | 27.2 |
| Ash, S ^39^ | 2006 | 31 | No intervention control | 3 | 35.8 |
| Assuncao, MCF ^40^ | 2010 | 95 | Usual care | 6 | 34.6 |
| Atlantis, E ^41^ | 2006 | 23 | Wait list control | 6 | 25.0 |
| Azadbakht, L ^42^ | 2005 | 40 | No intervention control | 6 | 29.5 |
| Balcazar, HG ^43^ | 2010 | 126 | Usual care | 4 | 31.1 |
| Belalcazar, LM ^44^ | 2010 | 836 | Usual care | 12 | 36.0 |
| Bouchard, DR ^45^ | 2009 | 12 | No intervention control | 3 | 32.3 |
| *Brown, C ^46^ | 2006 | 15 | No intervention control | 3 | 36.2 |
| Burke, V^47^ | 2005 | 98 | Usual care | 12 | 29.7 |
| Carroll, S ^48^ | 2007 | 10 | Wait list control | 3 | 37.9 |
| de Mello, VD ^49^ | 2008 | 10 | No intervention control | 8 | 31.6 |
| Dengo, AL ^20^ | 2010 | 11 | No intervention control | 3 | 31.8 |
| Eiben, G ^50^ | 2005 | 16 | Wait list control | 12 | 25.9 |
| Elliot, DL ^51^ | 2007 | 129 | Usual care | 12 | 27.9 |
| Eriksson, KM ^52^ | 2006 | 63 | Usual care | 12 | 29.4 |
| Faucher, MA ^53^ | 2010 | 8 | Usual care | 5 | 33.8 |
| Fenkci, S ^54^ | 2006 | 17 | No intervention control | 3 | 35.9 |
| Folta, SC ^55^ | 2009 | 30 | Wait list control | 3 | 32.1 |
| Ghroubi, S ^56^ | 2009 | 29 | No intervention control | 2 | 38.2 |
| *Gorin, AA ^57^ | 2008 | 169 | No intervention control | 12 | 30.1 |
| Groeneveld, IF ^58^ | 2010 | 256 | Usual care | 6 | 28.2 |
| Gutschall, MD ^59^ | 2009 | 48 | Wait list control | 2 | 33.4 |
| Haapala, I ^60^ | 2009 | 40 | Wait list control | 12 | - |
| Hara, T ^19^ | 2005 | 7 | No intervention control | 5 | 33.5 |
| Hardcastle, S ^61^ | 2008 | 131 | Usual care | 6 | 34.0 |
| Howard, BV ^62^ | 2006 | 25056 | Usual care | 12 | 29.1 |
| Hoy, MK ^63^ | 2009 | 1462 | Usual care | 12 | 27.5 |
| Ibanez, J ^64^ | 2009 | 9 | No intervention control | 4 | 35.0 |
| Jiang, X ^65^ | 2007 | 84 | Usual care | 3 | - |
| Johansson, K ^25^ | 2009 | 33 | Wait list control | 2 | 34.8 |
| Kattelmann, KK ^66^ | 2009 | 53 | Usual care | 6 | 34.3 |
| *Kim, KH ^67^ | 2008 | 34 | Wait list control | 2 | 34.7 |
| Kirkwood, L ^68^ | 2007 | 18 | No intervention control | 3 | 32.5 |
| Kosaka, K ^21^ | 2005 | 356 | Usual care | 48 | 23.8 |
| Lally, P ^69^ | 2008 | 33 | Wait list control | 2 | 29.5 |
| Ledikwe, JH ^70^ | 2007 | 223 | Usual care | 6 | 33.4 |
| Levine, MD ^71^ | 2007 | 75 | Usual care | 12 | 25.0 |
| Lockwood, CM ^72^ | 2008 | 10 | No intervention control | 3 | 26.5 |
| Lutes, LD ^73^ | 2008 | 19 | Wait list control | 4 | 31.8 |
| Mahon, AK ^74^ | 2007 | 11 | No intervention control | 2 | 30.1 |
| Masley, SC ^75^ | 2006 | 20 | No intervention control | 5 | 29.8 |
| McConnon, A ^76^ | 2007 | 77 | Usual care | 12 | 34.4 |
| McDoniel, SO (a) ^23^ | 2010 | 37 | Usual care | 6 | 35.4 |
| McDoniel, SO (b) ^77^ | 2010 | 41 | Usual care | 3 | 36.2 |
| McKibbin, CL ^78^ | 2006 | 29 | Usual care | 6 | 32.9 |
| Mefferd, K ^79^ | 2007 | 29 | Wait list control | 4 | 31.1 |
| Merrill, RM ^16^ | 2008 | 174 | No intervention control | 2 | 31.4 |
| Milano, W ^80^ | 2007 | 10 | No intervention control | 12 | 24.2 |
| Ng, TW ^24^ | 2010 | 15 | No intervention control | 4 | 33.4 |
| Oldroyd, JC ^81^ | 2006 | 24 | No intervention control | 6 | - |
| Paineau, DL ^82^ | 2008 | 393 | Usual care | 8 | 24.0 |
| Park, HA ^17^ | 2007 | 246 | Usual care | 54 | 25.0 |
| Pierce, GL ^83^ | 2008 | 14 | Usual care | 4 | 31.0 |
| Pimentel, GD ^26^ | 2010 | 24 | No intervention control | 12 | 28.0 |
| *Porsdal, V ^84^ | 2010 | 59 | No intervention control | 3 | 29.6 |
| *Poulin, MJ ^85^ | 2007 | 51 | No intervention control | 18 | 31.1 |
| Racette, SB ^86^ | 2006 | 10 | Usual care | 12 | 27.9 |
| Racette, SB ^87^ | 2009 | 55 | No intervention control | 12 | 31.1 |
| Rimmer, JH ^22^ | 2009 | 31 | Usual care | 12 | 43.6 |
| *Robbins, AS ^15^ | 2006 | 65089 | No intervention control | 12 | - |
| Rodearmel, SJ ^88^ | 2006 | 19 | No intervention control | 4 | 28.2 |
| Roumen, C ^89^ | 2008 | 54 | Usual care | 12 | 29.2 |
| Sallit, J ^18^ | 2009 | 58 | No intervention control | 3 | 28.1 |
| Samuel-Hodge, CD ^90^ | 2009 | 69 | Usual care | 12 | 35.1 |
| Sarsan, A ^91^ | 2006 | 20 | No intervention control | 3 | 25.5 |
| Sartorelli, DS ^92^ | 2005 | 53 | Usual care | 6 | 28.7 |
| Schwab, U ^93^ | 2008 | 19 | No intervention control | 8 | 34.2 |
| Stahre, L ^94^ | 2005 | 43 | Wait list control | 18 | 39.2 |
| Straznicky, NE ^95^ | 2010 | 19 | No intervention control | 3 | 33.0 |
| Subak, LL ^96^ | 2005 | 19 | Wait list control | 3 | 36.0 |
| Svendsen, M ^97^ | 2007 | 57 | Usual care | 3 | 37.6 |
| Thompson, CA ^98^ | 2005 | 31 | Usual care | 48 | 26.9 |
| Thoolen, BJ ^99^ | 2009 | 102 | Usual care | 3 | 29.7 |
| Toobert, DJ ^100^ | 2005 | 116 | Usual care | 6 | 35.6 |
| *Torres, SJ ^101^ | 2007 | 27 | No intervention control | 3 | 28.8 |
| Tully, MA ^102^ | 2005 | 9 | Wait list control | 3 | 27.3 |
| Tuomilehto, HP ^103^ | 2009 | 37 | Usual care | 12 | 31.4 |
| Vissers, D ^104^ | 2010 | 21 | No intervention control | 6 | 30.8 |
| von Gruenigen, VE ^105^ | 2008 | 16 | Usual care | 6 | 41.1 |
| Weber, M ^14^ | 2006 | 7 | Usual care | 4 | 33.0 |
| Weiss, EP ^106^ | 2008 | 9 | Usual care | 12 | 27.9 |
| Werkman, A ^107^ | 2010 | 154 | Usual care | 24 | 27.3 |

**Quasi-experimental trials*
